# Cholera toxin phage: structural and functional diversity between *Vibrio cholerae* biotypes

**DOI:** 10.3934/microbiol.2020009

**Published:** 2020-05-28

**Authors:** Ashrafus Safa, Jinath Sultana Jime, Farishta Shahel

**Affiliations:** 1 School of Environment and Life Sciences, Independent University, Bangladesh, Dhaka, Bangladesh; 2 Department of Biochemistry and Microbiology, School of Health and Life Sciences, North South University, Dhaka, Bangladesh

**Keywords:** *Vibrio cholerae*, classical, El Tor, cholera toxin phage, cholera toxin, cholera, RS1, *ctxA*, *ctxB*, *rstR*

## Abstract

Cholera is a severe form of watery diarrhea caused by *Vibrio cholerae* toxigenic strains. Typically, the toxigenic variants of *V. cholerae* harbor a bacteriophage, cholera toxin phage, integrated in their genome. The *ctxAB* genes from the phage genome encode the cholera toxin, which is responsible for the major clinical symptoms of the disease. Although *ctxAB* genes are crucial to *V. cholerae* strains for cholera manifestation, the genetic structure of cholera toxin phage, DNA sequence of its genes, spatial organization in the host genome and its satellite phage content are not homogenous between *V. cholerae* biotypes—classical and El Tor. Differences in cholera toxin phage and its genes play a significant role in the identification of *V. cholerae* biotypes and in the understanding of their pathogenic and epidemic potentials. Here, we present an account of the variations of cholera toxin phage and its genes in *V. cholerae* biotypes as well as their usefulness in the identification of classical and El Tor strains.

## 1. Introduction

*Vibrio cholerae* is an ancient human pathogen that causes a severe form of diarrhea known as cholera. Humans are the only medically relevant living host of *V. cholerae*, and its transmission is mainly mediated by the consumption of *V. cholerae*-contaminated water and/or food. Endemic cholera in underdeveloped and developing countries frequently gives rise to explosive outbreaks that sometimes result in pandemics. During the course of recorded human history, *V. cholerae* has caused seven pandemics [1],[2].

Cholera is characterized by copious watery stools that resembles rice water and by vomiting. A filamentous bacteriophage, known as ‘Cholera Toxin Phage’ (CTXφ), integrates into the genome of *V. cholerae* strains and enables them to produce Cholera toxin (CT)—the key virulence factor responsible for the clinical manifestations of cholera. The CT, encoded by *ctxA* and *ctxB* genes, is an A-B type toxin comprising A and B subunits. CtxA (or CTA) comprises of two polypeptide chains, CtxA1 and CtxA2, of which A1 provides CT-mediated toxigenicity, while A2 possibly affords a linker function and appends A1 to CtxB. The CtxB (or CTB) subunit is a homopentamer and is responsible for CT binding to the ganglioside GM1 of intestinal epithelium [3]. After binding to GM1, CT is endocytosed by the cell. For cell intoxication to occur, the entry of CTA1 to the cell cytosol is the key step because CTA1 catalyzes the ADP ribosylation of the adenylate cyclase (AC) [3]. ADP-ribosylation resulted in enhanced AC activity and an increased intracellular cAMP concentration, which in turn produce an imbalance in electrolyte movement in the epithelial cell and develop cholera.

Based on variations in the somatic O-antigen structure, until now >209 serogroups of *V. cholerae* have been identified. Of these, 95% or more of *V*. *cholerae* strains belonging to serogroup O1 and O139 are toxigenic i.e., they harbor CTXφ and have the ability to produce CT. Based on the biochemical and phenotypic differences among *V. cholerae* strains from serogroup O1, two clearly distinct biotypes (a nominate form or subspecies), ‘classical’ and ‘El Tor’, have been described. The typical responses of classical strains to conventional biochemical and phenotypic tests show a negative Voges-Proskauer (VP) reaction (acetoin production), no chicken cell (erythrocyte) agglutination (CCA), sensitivity to Polymyxin B and phage IV, and resistance to phage 5, while El Tor strains are positive for VP reaction and CCA, resistant to Polymyxin B and phage IV, but sensitive to phage 5 [1]. Notably, the sixth and presumably the fifth pandemics were caused by classical biotype. By contrast, the ongoing seventh pandemic that started in 1961 was caused by El Tor biotype of *V. cholerae* O1. In addition to the phenotypic differences, structure of CTXφ and its genes in classical and El Tor strains vary significantly and these differences have long been used to differentiate *V. cholerae* biotypes. For example, based on the differences in *ctxB* DNA sequences, ‘*ctxB* genotyping’ scheme was developed to differentiate *V. cholerae* biotypes [4]. Additionally, CTXφs are also known to differ in their arrangement, their choice of host chromosome type (small and/or large) for integration, and their copy number in each chromosome of the strains of the two biotypes. In this review, we summarize our knowledge of the structural and functional diversity of CTXφ and its genes in the genome of classical and El Tor strains and their applications in differentiating these two biotypes.

## 2. Structural and functional diversity of CTX prophage in *V. cholerae* biotypes

CTXφ is an atypical filamentous phage that carries some of the most critical virulence genes of *V. cholerae* toxigenic strains [5],[6]. Toxin co-regulated pilus (TCP) is a type IV pilus that acts as the receptor for CTXφ virions and is an essential human intestinal colonization factor for *V. cholerae* strains [1]. Once the CTXφ is inside the host cell, it can either lysogenize *V. cholerae* strains by integrating into the host chromosome at the *attRS* attachment site or replicate episomally inside the host [7]. Integration of the phage chromosome into the host genome is mediated by phage-encoded proteins (see below) in conjunction with the *V. cholerae*-encoded *xerC* and *xerD* gene products [8]. The primary function of XerC and XerD recombinases is to convert chromosome dimers into monomers by catalyzing recombination between sequences, known as *dif* sites, near the chromosomal termini [9]. Since CTXφ is maintained as an integrated prophage, production of virions requires production of extrachromosomal phage DNA. Unlike other filamentous phages, extrachromosomal CTXφ is not simply formed by prophage excision rather it is generated by a replicative process that depends upon the presence of tandemly arranged CTXφ and RS1φ (see below) within the chromosome. The advantage of this replicative process is that CTXφ can be transmitted to a new host and simultaneously retained within the genome of its old host. CTXφ virions are then secreted by their hosts using type IV secretion system [9] and infect new strains.

**Figure 1.**
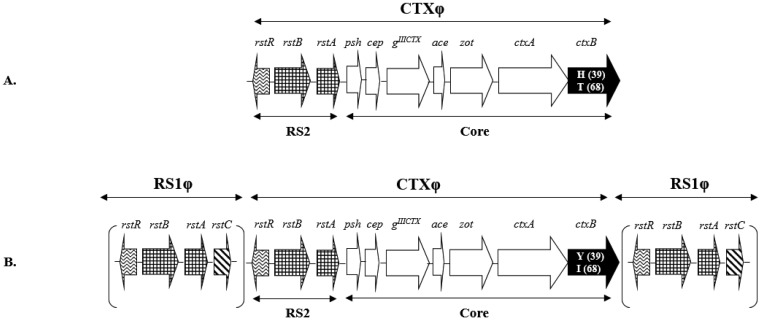
Typical genetic organization of the CTXφ in selected strains of *V. cholerae* biotypes. (A): CTXφ in the genome of 569B strain of classical biotype. (B): CTXφ in the genome of N16961 strain of El Tor biotype. The numbers in parentheses within the *ctxB* gene represent the amino acid substitution positions (where H, T, Y and I represent Histidine, Threonine, Tyrosine and Isoleucine, respectively). The RS1φ flanking the integrated CTXφ in El Tor are bracketed. Slash sign indicates alternate combination.

The single-stranded DNA phage genome is ∼7.0 kb long and consists of two regions: RS2 and the core domain (Figure 1A). Four of the five genes in the core domain, namely *cep*, *g^IIICTX^*, *ace* and *zot*, are associated with phage morphogenesis and accessory toxigenicity [10]–[12], whereas the function of the *ctxAB* genes in the phage morphogenesis is yet unknown. The 2.4-kb RS2 region comprises three genes: *rstA*, *rstB* and *rstR*, which encode replication, integration and regulatory functions, respectively, in the phage [7] (Figure 1A).

Structurally the core region of the CTXφ is identical in both classical and El Tor strains. Although a satellite phage, named RS1 (described in section 4), may or may not situated along either side of the CTX prophage in El Tor strains (Figure 1B) but CTXφ in classical strains were never known to flank by RS1φ. Sequence composition of *rstR* gene of the RS2 region of CTXφ were found to be highly divergent among various *V. cholerae* O1 strains. DNA sequence heterogeneity of the *rstR* gene laid the basis for the diverse CTXφ phage types in different *V. cholerae* strains including the two biotypes. For example, CTXφ in classical strains that harbor the classical-specific *rstR* allele (*rstR^classical^*) is designated as classical CTXφ (CTXφ^classical^), whereas the CTXφ in El Tor strains with the El Tor-specific *rstR^El Tor^* allele is designated as El Tor CTXφ (CTXφ^El Tor^) [13]. Two other CTXφ types, which are related to the *rstR^calcutta^* and *rstR^Environmental^* alleles, have been reported in *V. cholerae* O139 and environmental strains of diverse serotypes [13]–[16]. Interestingly, *V. cholerae* strains infrequently harbor multiple copies of different *rstR* genes, which indicate the presence of multiple copies of CTXφ or RS1 satellite (described in section 3) phages in the host genome [15],[17]. Apart from *rstR* gene, sequence composition of the *ctxB* gene varies significantly between *V. cholerae* biotypes (described in section 5).

Functional studies have revealed that repression of the *rstA* gene by the *rstR* allele is biotype-specific, which indicated that sequence diversity in the *rstR* gene provides the molecular basis for heteroimmunity, by which *V. cholerae* strains are immune to secondary infection by an identical CTXφ type [18]. The *rstR*-mediated biotype-specific heteroimmunity has been shown to protect El Tor strains harboring CTXφ^El Tor^ from further infection by the same strain, but not from infection with CTXφ^classical^ [18]. Heteroimmunity cannot be demonstrated in classical strains, since they do not produce virions, whereas El Tor strains are capable of generating both replicative and infectious phage particles, which is another factor used in discriminating classical and El Tor variants. However, exogenous replicative forms of CTXφ^classical^ have been rarely observed in classical strains that were presumed to be defective in phage replication [19],[20].

## 3. Diversity in CTXφ arrangement inside the genome of classical and El Tor biotypes

The spatial organization of CTXφ in the *V. cholerae* genome varies between the biotypes, with one or more copies of CTXφ integrating into a susceptible *V. cholerae* strain. It is known that El Tor strains normally harbor one or more CTXφ copies exclusively in the large chromosome; if two copies are present then they are arranged in tandem. In classical strains, one or both chromosomes can harbor CTXφ, which are usually dispersed [21],[22]. The molecular basis for CTXφ virion production in *V. cholerae* strains has been shown to rely on both the total CTXφ content and their spatial arrangement in the *V. cholerae* genome. In El Tor strains, the presence of multiple CTXφ copies in tandem (CTXφ-CTXφ) or a single CTXφ copy linked to RS1Ø (CTXφ-RS1φ) (Figure 1B) in *V. cholerae* genome was found to activate CTXφ replication to generate infectious virions. However, this pattern of CTXφ organization has never been reported in classical strains and therefore, classical strains were claimed to possess dysfunctional CTXφ with the inability to produce infectious virions [19]. Notably, a group of El Tor strains isolated just before the O139 outbreak in India in 1992 [23] and another group of El Tor-like strains from Mozambique were reported to harbor tandem arrays of CTXφ^classical^ exclusively in the small chromosome [20],[24], and yet these strains do not produce infectious CTXφ virions [20].

## 4. Biotype-specific differences in the RS1 satellite prophage of CTXφ

RS1 element is in fact the genome of a satellite phage (phages that do not encode their own structural components but rely on the bacterial host and another helper prophage for replication survival) that utilizes CTXφ morphogenesis genes to produce RS1φ particles [25]. However, CTXφ-independent mechanism of RS1φ has been reported in a nontoxigenic environmental *V. cholerae* strain [26]. CTXφ prophages in *V. cholerae* strains are usually flanked on either side by the RS1φ (Figure 1B). RS1 is very similar to the RS2 gene cluster, except that it contains an additional gene, *rstC*. RstC is an antirepressor that controls CTXφ lysogeny, production of CTXφ particles, and expression of cholera toxin [27]. RS1φ is biotype-specific and it is present in most, if not all, El Tor biotype strains examined to date [28] whereas no strains of classical biotype are known to harbor RS1φ. In El Tor strains, RS1 influences the phage virion production in two ways: one is creating an array of repeat sequences by flanking the CTXφ genome (see section 3) and another is upregulating CTXφ production by using RstC to inactivate the RstR that downregulate the CTXφ production [27].

## 5. Variation of *ctxB* gene sequence between classical and El Tor biotypes

**Figure 2.**
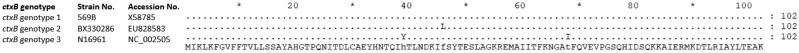
Alignment of deduced amino acid sequences of CtxB subunit found in *V. cholerae* biotypes. H, F, T, Y, L and I at positions, 39, 46 and 68 stand for amino acids Histidine, Phenylalanine, Threonine, Tyrosine, Leucine and Isoleucine, respectively.

Heterogeneity in *ctxB* gene sequence and variation in the immunological properties of CTB (encoded by *ctxB*) have long been utilized to differentiate *V. cholerae* variants. Based on epitope analysis of CTB, two immunologically related but not identical epitypes have been described. Epitype CT1 is elaborated by classical biotype strains and the US Gulf Coast El Tor strains, while CT2 is produced by the El Tor biotype and O139 strains [29]. Aside from epitope mapping, Olsvik et al. [4] in 1996, based on point mutations in *ctxB*, identified three *ctxB* genotypes by using automated DNA sequencing. These *ctxB* genotypes are known as genotype 1 (*ctxB1*), genotype 2 (*ctxB2*) and genotype 3 (*ctxB3*). In all these genotypes, three point mutations at positons 115 (C or T), 138 (T or G) and 203 (C or T) in *ctxB* gene [6] resulted in the deduced amino acid substitutions at positions 39 (histidine or tyrosine), 46 (phenylalanine or leucine) and 68 (threonine or isoleucine) in CTB subunit (Figure 2). Based on the *ctxB* genotyping method, the classical biotype and US Gulf Coast El Tor indigenous strains have been classified as genotype 1, the Australian indigenous *V. cholerae* El Tor strains as genotype 2, and the current seventh pandemic El Tor strains and the Latin American epidemic strains as genotype 3 [4]. Thus, in effect, *V. cholerae* classical strains of epitype 1/genotype 1 produce classical CT (CT^classical^), while the El Tor biotype strains of epitype 2/genotype 3 produce El Tor CT (CT^El Tor^). However, current investigation has revealed that additional *ctxB* alleles exist among *V. cholerae* strains and until now, nine *ctxB* genotypes have been recognized [28]. Notably, differences in the functional aspects of these *ctxB* alleles are yet to be revealed.

## 6. Final remarks and future perspective

The evolution of pathogenic variants seems to be a natural phenomenon that occurs in microbes and other forms of life in response to an ever-changing environment, and *V. cholerae* is no exception. The significance of CTXφ and its genes in the evolution of pathogenic form of *V. cholerae* is immense and indisputable. Therefore, continuous monitoring of the changes in the CTX prophage genome among *V. cholerae* variants including classical and El Tor strains could be one of the most effective ways to track the emergence of new CTXφ variants, therefor the *V. cholerae* strains. Furthermore, early detection of the emerging variants would facilitate formulating better control measures including vaccine development.

## References

[1] Kaper JB, Morris JG, Levine MM (1995). Cholera. Clin Microbiol Rev.

[2] Albert M, Ansaruzzaman M, Bardhan P (1993). Large epidemic of cholera-like disease in Bangladesh caused by *Vibrio cholerae* 0139 synonym Bengal. The Lancet.

[3] Sánchez J, Holmgren J (2008). Cholera toxin structure, gene regulation and pathophysiological and immunological aspects. Cell Mol Life Sci.

[4] Olsvik Ø, Wahlberg J, Petterson B (1993). Use of automated sequencing of polymerase chain reaction-generated amplicons to identify three types of cholera toxin subunit B in Vibrio cholerae O1 strains. J Clin Microbiol.

[5] Mekalanos JJ (1985). Cholera toxin: genetic analysis, regulation and role in pathogenesis. Curr Top Microbiol Immunol.

[6] Waldor MK, Mekalanos JJ (1996). Lysogenic conversion by a filamentous bacteriophage encoding cholera toxin. Science.

[7] Waldor MK, Rubin EJ, Pearson GDN (1997). Regulation, replication, and integration functions of the *Vibrio cholerae* CTXφ are encoded by regions RS2. Mol Microbiol.

[8] Huber KE, Waldor MK (2002). Filamentous phage integration requires the host recombinases XerC and XerD. Nature.

[9] Davis BM, Waldor MK (2003). Filamentous phages linked to virulence of *Vibrio cholerae*. Curr Opin Microbiol.

[10] Trucksis M, Galen JE, Michalski J (1993). Accessory cholera enterotoxin (Ace), the third toxin of a *Vibrio cholerae* virulence cassette. Proc Natl Acad Sci USA.

[11] Faruque SM, Albert MJ, Mekalanos JJ (1998). Epidemiology, genetics, and ecology of toxigenic *Vibrio cholerae*. Microbiol Mol Biol Rev.

[12] Heilpern AJ, Waldor MK (2003). pIIICTX, a predicted CTXφ minor coat protein, can expand the host range of coliphage fd to include *Vibrio cholerae*. J Bacteriol.

[13] Kimsey HH, Nair GB, Ghosh A (1998). Diverse CTXφ and evolution of new pathogenic *Vibrio cholerae*. Lancet.

[14] Davis BM, Kimsey HH, Kimsey WC (1999). The *Vibrio cholerae* O139 Calcutta bacteriophage CTXφ is infectious and encodes a novel repressor. J Bacteriol.

[15] Mukhopadhyay AK, Chakaraborty S, Takeda Y (2001). Characterization of VPI pathogenicity island and CTXφ prophage in environmental strains of *Vibrio cholerae*. J Bacteriol.

[16] Bhattacharya T, Chatterjee S, Maiti D (2006). Molecular analysis of the *rstR* and *orfU* genes of the CTX prophages integrated in the small chromosomes of environmental *Vibrio cholerae* non-O1, non-O139 strains. Environ Microbiol.

[17] Nusrin S, Khan GY, Bhuiyan NA (2004). Diverse CTX phages among toxigenic *Vibrio cholerae* O1 and O139 strains isolated between 1994 and 2002 in an area where cholera is endemic in Bangladesh. J Clin Microbiol.

[18] Kimsey H, Waldor MK (1998). CTXφ immunity: application in the development of cholera vaccines. Proc Natl Acad Sci USA.

[19] Davis MB, Moyer KE, Boyd EF (2000). CTX prophages in classical biotype *Vibrio cholerae*: functional phage genes but dysfunctional phage genomes. J Bacteriol.

[20] Faruque SM, Tam VC, Chowdhury N (2007). Genomic analysis of the Mozambique strain of *Vibrio cholerae* O1 reveals the origin of El Tor strains carrying classical CTX prophage. Proc Natl Acad Sci USA.

[21] Mekalanos JJ (1983). Duplication and amplification of toxin genes in *Vibrio cholerae*. Cell.

[22] Trucksis M, Michalsky J, Deng YK (1998). The *Vibrio cholerae* genome contains two unique circular chromosomes. Proc Natl Acad Sci USA.

[23] Nandi S, Maiti D, Saha A (2003). Genesis of variants of *Vibrio cholerae* O1 biotype El Tor: role of the CTXφ array and its position in the genome. Microbiology.

[24] Lee JH, Han KH, Choi SY (2006). Multilocus sequence typing (MLST) analysis of *Vibrio*
*cholerae* O1 El Tor isolates from Mozambique that harbour the classical CTX prophage. J Med Microbiol.

[25] Faruque SM, Asadulghani, Kamruzzaman M (2002). RS1 element of *Vibrio cholerae* can propagate horizontally as a filamentous phage exploiting the morphogenesis genes of CTXphi. Infect Immun.

[26] Faruque SM, Kamruzzaman M, Asadulghan (2003). CTXφ-independent production of the RS1 satellite phage by Vibrio cholerae. Proc Natl Acad Sci USA.

[27] Davis BM, Kimsey HH, Kane AV (2002). A satellite phage-encoded antirepressor induces repressor aggregation and cholera toxin gene transfer. EMBO J.

[28] Safa A, Nair GB, Kong RYC (2010). Evolution of new variants of *Vibrio cholerae* O1. Trends Microbiol.

[29] Finkelstein RA, Burks F, Zupan A (1987). Epitopes of the cholera family of enterotoxins. Rev Infect Dis.

